# Early cases of SARS-CoV-2 infection in Uganda: epidemiology and lessons learned from risk-based testing approaches – March-April 2020

**DOI:** 10.1186/s12992-020-00643-7

**Published:** 2020-11-25

**Authors:** Richard Migisha, Benon Kwesiga, Bernadette Basuta Mirembe, Geofrey Amanya, Steven N. Kabwama, Daniel Kadobera, Lilian Bulage, Godfrey Nsereko, Ignatius Wadunde, Tonny Tindyebwa, Bernard Lubwama, Atek A. Kagirita, John T. Kayiwa, Julius J. Lutwama, Amy L. Boore, Julie R. Harris, Henry Kyobe Bosa, Alex Riolexus Ario

**Affiliations:** 1 Uganda Public Health Fellowship Program, Ministry of Health, Kampala, Uganda; 2 Ministry of Health, Kampala, Uganda; 3 Makerere University School of Public Health, Kampala, Uganda; 4 Uganda Virus Research Institute, Entebbe, Uganda; 5 US Centers for Disease Control and Prevention, Kampala, Uganda; 6 Uganda People’s Defence Forces, Kampala, Uganda

**Keywords:** COVID-19, SARS-Cov-2, Epidemiology, Surveillance, Uganda

## Abstract

**Background:**

On March 13, 2020, Uganda instituted COVID-19 symptom screening at its international airport, isolation and SARS-CoV-2 testing for symptomatic persons, and mandatory 14-day quarantine and testing of persons traveling through or from high-risk countries. On March 21, 2020, Uganda reported its first SARS-CoV-2 infection in a symptomatic traveler from Dubai. By April 12, 2020, 54 cases and 1257 contacts were identified. We describe the epidemiological, clinical, and transmission characteristics of these cases.

**Methods:**

A confirmed case was laboratory-confirmed SARS-CoV-2 infection during March 21–April 12, 2020 in a resident of or traveler to Uganda. We reviewed case-person files and interviewed case-persons at isolation centers. We identified infected contacts from contact tracing records.

**Results:**

Mean case-person age was 35 (±16) years; 34 (63%) were male. Forty-five (83%) had recently traveled internationally (‘imported cases’), five (9.3%) were known contacts of travelers, and four (7.4%) were community cases. Of the 45 imported cases, only one (2.2%) was symptomatic at entry. Among all case-persons, 29 (54%) were symptomatic at testing and five (9.3%) were pre-symptomatic. Among the 34 (63%) case-persons who were ever symptomatic, all had mild disease: 16 (47%) had fever, 13 (38%) reported headache, and 10 (29%) reported cough. Fifteen (28%) case-persons had underlying conditions, including three persons with HIV. An average of 31 contacts (range, 4–130) were identified per case-person. Five (10%) case-persons, all symptomatic, infected one contact each.

**Conclusion:**

The first 54 case-persons with SARS-CoV-2 infection in Uganda primarily comprised incoming air travelers with asymptomatic or mild disease. Disease would likely not have been detected in these persons without the targeted testing interventions implemented in Uganda. Transmission was low among symptomatic persons and nonexistent from asymptomatic persons. Routine, systematic screening of travelers and at-risk persons, and thorough contact tracing will be needed for Uganda to maintain epidemic control.

## Introduction

COVID-19, the respiratory disease caused by severe acute respiratory syndrome coronavirus 2 (SARS-CoV-2) was first reported in Wuhan, China in late December 2019 [1]. On January 30, 2020, SARS-CoV-2 was declared a public health emergency of international concern, having spread to multiple countries outside of China [2]. By March 11, 2020, it was declared a global pandemic, with approximately 118,000 confirmed cases and nearly 4300 deaths on all continents except Antarctica [3]. However, Uganda had not yet reported any cases of SARS-CoV-2 infection.

On March 13, 2020, having noted the rapid spread of SARS-CoV-2 in most other countries in the world, Uganda instituted multiple measures to prevent entry and spread. These included symptom screening at the airport, isolation and testing for symptomatic persons, and a mandatory 14-day institutional quarantine and testing of travelers from high-risk countries [4]. Persons entering from low-risk countries [4] were asked to self-quarantine but were not tested unless they had symptoms on arrival. Travelers in quarantine were tested if they developed symptoms or on Day 14 of quarantine, regardless of symptoms. Effective March 23, 2020, the country implemented a ban on all international travel, and closed both schools and universities. One day later, the Ministry of Health requested all travelers entering Uganda the United Arab Emirates in the past two weeks to self-present for testing. Subsequently, all persons who had traveled from any international destination into Uganda since March 7 were asked to self-present for testing. On March 30, 2020, the country instituted a complete lockdown, banning all public transport and public gatherings.

On March 21, 2020, Uganda confirmed its first SARS-CoV-2 infection in a Ugandan traveler from the United Arab Emirates. By April 15, 2020, 54 cases had been reported in country [5]. The country implemented thorough contact tracing for all infected persons. We describe the interventions in place and the characteristics of and transmission from the first 54 case-persons to provide guidance for near-future interventions against SARS-CoV-2 spread in Uganda.

## Methods

### Case definition and study procedures

**Fig. 1:**
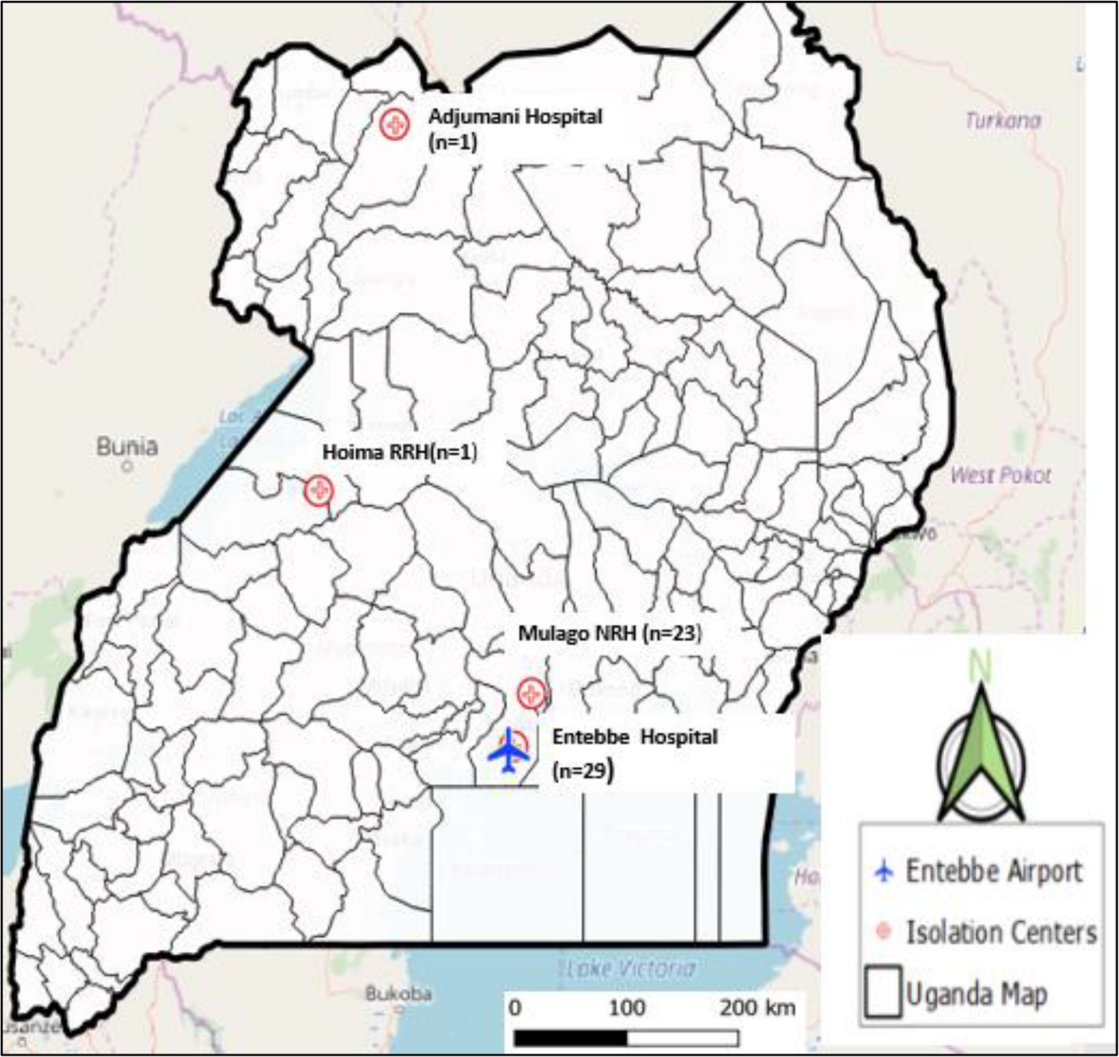
Hospitals that managed the initial 54 persons with SARS-CoV-2 infection in Uganda, March–April, 2020 (map drawn using QGIS browser 3.10.2)

We defined a confirmed case as laboratory-confirmed SARS-CoV-2 infection identified during March 21–April 12, 2020 in a resident of or traveler to Uganda. A suspected case was defined as onset of acute respiratory illness with high temperature (above 37.5 °C) and at least one sign/symptom of respiratory illness (e.g., cough, shortness of breath), AND a history of travel to or residence in a location reporting community transmission of SARS-CoV-2 during the last 14 days prior to symptom onset, in a resident or traveler to Uganda from March 21–April 12, 2020. Laboratory testing of oropharyngeal and/or nasopharyngeal swabs was done at Uganda Virus Research Institute (UVRI) using real-time RT-PCR (Berlin protocol) [6]. All confirmed case-persons were isolated in hospitals. We reviewed case files and interviewed all case-persons at isolation centers located within four hospitals treating case-persons: Mulago National Referral Hospital, Entebbe Grade B Hospital, Adjumani General Hospital, and Hoima Regional Referral Hospital (Fig. 1). All case-persons were tested for HIV on entry and were asked about other underlying diseases. We also conducted a telephone interview with one case-person who was isolated and being managed at home.

### Assessment of symptoms

We assessed for presence of symptoms during the 14 days preceding sample collection/testing. All case-persons were subsequently monitored for development of new symptoms while admitted in hospital using a self-monitoring tool for symptoms. We categorized case-persons as symptomatic if they had had any new or worsened typical or atypical COVID-19 symptom in the previous 14 days. We classified case-persons as having typical symptoms if they had temperature greater than 37.5 °C (99.5 °F) or self-reported fever, shortness of breath, or cough [7]. We categorized case-persons as having atypical symptoms if they had at least one of the following: chills, confusion/irritability, body weakness, nasal congestion, runny nose, sore throat, joint pains, muscle pains, chest pain, dizziness, headache, abdominal pain, nausea, or diarrhea without any of the typical symptoms [7]. We classified case-persons as asymptomatic if they had no new symptoms or no worsening chronic symptoms (e.g., stable chronic cough). Presymptomatic case-persons were those who were asymptomatic at the time of sample collection but developed symptoms within the 14 days of isolation/admission. Case-persons who did not develop any new symptoms in the 14 days after isolation were still classified as asymptomatic.

### Tracing of contacts

Contacts were defined as persons with face-to-face contact (< 2 m) with a case-person or persons who spent time in a closed environment with a case-person, including coworkers or household members; healthcare workers or other persons providing direct care for case-persons without recommended personal protective equipment; persons sitting within 5 seats in any direction from the case-person in a vehicle or plane; or crew working in the section of the plane or vehicle where a case-person was seated. For symptomatic case-persons, all persons exposed to the case-person from two days before to 14 days after the case-person’s symptom onset were listed as contacts as per Ministry of Health guidelines. For asymptomatic case-persons, all contacts exposed to the case-person in the 14 days before the case-person’s laboratory confirmation were listed as contacts. This was done to account for our inability to identify the infectious period for asymptomatic case-persons. Contacts were initially tested just before release from quarantine, or any time they developed symptoms; however, guidance changed during the period to include a test midway through quarantine as well. We reviewed contact tracing records to identify the number of contacts per case.

### Statistical analyses

Data were entered and analyzed in Epi Info 7.2. Software (CDC, Atlanta, USA). We described method of case-person identification as well as epidemiologic, demographic, and clinical characteristics of case-persons. Continuous variables were presented as means (SD) if they were normally distributed or medians (IQR) if they were not, and categorical variables as frequencies (%). The clinical outcomes of the case-persons were described through April 12, 2020. For asymptomatic cases, we considered date of sample collection as a proxy for date of illness onset.

## Results

A total of 5025 tests for SARS-CoV-2 had been carried out in Uganda as of April 12, 2020. Of these, samples from 54 (1.1%) persons were positive for SARS-CoV-2. From these 54 case-persons, 1257 contacts were identified. A total of 1105 high-risk travelers had been placed in institutional quarantine as of April 12, 2020, of whom 861 (78%) had been discharged by that date.

### Demographics

The mean age of the case-persons was 35 (±16) years, with a range of 7–66 years (Table 1); 34 (63%) were male. Most (91%) were Ugandan and 45 (83%) had history of international travel, mainly to United Arab Emirates and United Kingdom (Table 1). Of 37 case-persons who shared their occupation, 19 (51%) were in business (mainly dealing with imports/exports).

**Table 1:** Demographic characteristics of first 54 persons with SARS-CoV-2 infection admitted to hospitals in Uganda

Characteristic	Total (***N*** = 54)	%
	***n***	
**Age**^**a**^ **category**		
0–9 Years	4	7
10–19 Years	4	7
20–29 Years	11	20
30–39 Years	18	33
40–49 Years	6	11
50–59 Years	6	11
60 years & above	8	15
**Male Sex**	34	63
Ugandan	39	91
Chinese	2	4
Congolese	1	2
Canadian	1	2
**Resident in Uganda**	**51**	**95**
**Occupation**		
Business	19	35
Employed/civil servant	6	11
Transporter	3	6
Farmer	2	4
Student	1	2
Others	6	11
Not disclosed	17	31
**Marital status**		
Single	25	46
Married/Living with partner	29	54
**Contact with confirmed COVID-19 case**	**17**	**32**
**International travel history (Imported cases)**	**45**	**83**
United Arab Emirates	18	40
United Kingdom	14	31
Turkey	2	4
USA	2	4
Other countries^b^	9	20

^a^ Mean (SD) age of 35 (±15.9) years

^b^One person each traveled to Canada, China, Afghanistan, South Sudan, Kenya, Rwanda, Ethiopia, Germany, and The Netherlands

### Clinical characteristics of case-persons

Among the 34 case-persons who eventually developed symptoms, 47% reported fever, 38% reported headache, and 29% reported cough (Table 2). Among all 34 symptomatic case-persons, only one (3%) had all three typical symptoms of COVID-19 (Table 2). None of the case-persons developed severe disease requiring supplemental oxygen, intensive care, or life support, and none died.

**Table 2:** Clinical characteristics of first 54 persons with SARS-CoV-2 infection admitted to hospitals in Uganda

Characteristic (***n*** = 54 cases)	***n***	%
**Onset of symptoms**		
Symptomatic at sample collection	29	54
Became symptomatic after sample collection	5	9
Never became symptomatic at all	20	37
**Had underlying medical condition**	15	28
**Comorbidities**^**c**^ **(*n*** **= 15)**		
Hypertension	6	40
Diabetes mellitus	4	27
HIV	2	13
Pre-existing malignancy	1	7
HIV & diabetes mellitus	1	7
Hypertension & diabetes mellitus	1	7
**Pregnant**	1	2
**Symptoms (*n*** **= 34)**		
Fever^a^	16	47
Headache	13	38
Cough^ab^	10	29
Runny nose^b^	8	23
Chest pain^b^	6	18
Chills	4	12
Shortness of breath^ab^	3	9
Sore throat^b^	3	9
Nausea	3	9
Diarrhe	3	9
Joint pains	2	6
Irritability or confusion	2	6
Vomiting	1	3
Abdominal pain	1	3
**Symptom classification (*n*** **= 34)**		
Any one typical^a^ symptom	22	65
Two typicala symptoms	6	18
All three typical^a^ symptoms	1	3
Any respiratory^b^ symptoms	16	47
Only atypical symptoms	12	35
**Treatment outcome at time of manuscript-writing**		
Recovered	46	85
Still in isolation	8	15
Admitted to intensive care unit	0	0
Died	0	0

^a^Typical symptoms defined as: fever, cough, shortness of breath [7]. ^b^Respiratory symptoms defined as: cough, chest pain, shortness of breath, runny nose and sore throat;

^c^Comorbidities: *HIV* Hypertension, Diabetes mellitus, Malignancy

Approximately three-quarters of the case-persons had no known underlying illnesses. Among 15 (28%) case-persons with underlying disease, six had hypertension alone, four had diabetes alone, two had HIV alone, and one had a pre-existing malignancy. In addition, one patient had both HIV and diabetes, and one had hypertension and diabetes. One case-person was pregnant in her third trimester at the time she was diagnosed with COVID-19 (Table 2).

### Case-person identification by location

**Fig. 2:**
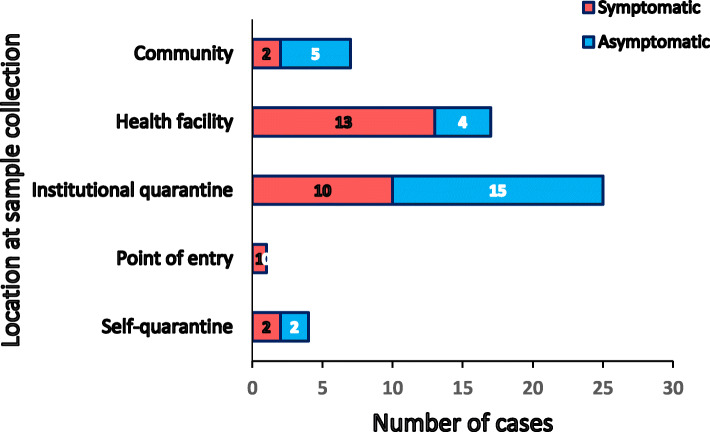
Location of case-persons with SARS-CoV-2 infection at the time of sample collection, by symptom status, Uganda, 2020

Of the 54 case-persons, 45 (83%) were persons who had traveled in the past 14 days. Among these, one was symptomatic and identified during airport screening when he entered the country (from Dubai), and had been sent directly from the airport to a testing site (Fig. 2).

An additional 25 were sent to institutional quarantine after returning from countries that had been categorized by Uganda as high-risk. Of these, 10 (40%) case-persons became symptomatic while in quarantine and were tested as a result, while 15 (60%) were identified during routine testing of asymptomatic persons in institutional quarantine. All remaining 19 travelers had come in from countries categorized as low-risk [4] and self-presented following a request from the Uganda Ministry of Health that recent travelers present for testing. Ten (50%) of the travelers who tested positive after this request came from the United Arab Emirates.

Among the nine case-persons who did not travel, five were identified through contact tracing in the community; two (40%) were symptomatic at the time of identification. The remaining four were identified at the health facility as part of routine screening of those who met definition for suspected COVID-19 case. Most of the case-persons were residents of the Kampala capital city (43%) and its periurban neighboring district, Wakiso (24%).

### Description by time

**Fig. 3:**
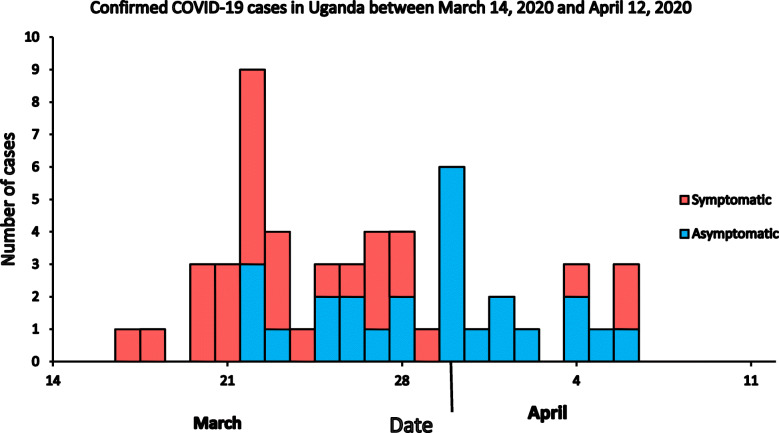
The initial 54 persons with SARS-CoV-2 infection in Uganda by date. Red bars represent symptom onset dates for symptomatic case-persons. Blue bars represent the specimen collection date for asymptomatic case-persons

Illness onsets ranged from March 17 to April 6, 2020, with the highest number of case-persons having illness onset (or sample-taking, for asymptomatic patients) on March 22, 2020 (Fig. 3).

### Contact tracing

Contact tracing was carried out for all case-persons. Of the 1257 contacts identified as of April 12, 2020, 1244 (99%) were followed up. Of these, 603 (49%) had completed the mandatory 14-day follow-up period and had been discharged from daily follow up. An average of 31 contacts (range, 4–130) were identified for each case-person. Contacts were tested for SARS-CoV-2 a mean of 1.3 times (range, 1–3 times); five contacts tested positive.

### Transmission dynamics among initial cases in Uganda

Among 20 asymptomatic case-persons, no forward-transmission chains within Uganda were identified. Among 34 symptomatic case-persons, five (15%) transmitted infection to a single person each. Nine (17%) of the 54 case-persons spent between 1 and 12 (median: 4) days symptomatic in the Ugandan community before isolation, and of these, three (33%) transmitted infection. Infection was also transmitted from two case-persons to others after developing symptoms in quarantine. In a single cluster of 11 case-persons, transmission could not be conclusively stated to have occurred in Uganda, as all had been traveling together previously in the United Kingdom and had traveled back Uganda on the same flight. All had become ill within one incubation period of travel (travel back to Uganda on March 20, 2020; onsets on March 20–March 31, 2020). Four of the confirmed case-persons, all of whom were symptomatic and identified as suspected cases at presentation at health facilities, had no history of travel and no known contact with a confirmed case.

## Discussion

We describe the epidemiological characteristics of the initial 54 persons with confirmed SARS-CoV-2 infection in Uganda. Most (83%) cases were imported, had atypical symptoms or few symptoms, with nearly half of patients being asymptomatic. Many of the early cases in Uganda, including the index case, were in persons who had traveled to countries not previously known to be high-risk [4], and were therefore not required to be in institutional quarantine. Despite extensive contact tracing and testing of contacts, few transmissions were identified.

Other countries have reported lower proportions of asymptomatic patients among their initial cases [8–13]. The high proportion of asymptomatic cases in Uganda is undoubtedly due to the national strategy, implemented on March 13, 2020, of testing all incoming travelers from high-risk countries and subsequently recalling and testing travelers from all countries, regardless of symptoms. As one of the last countries to be affected by COVID-19 globally, Uganda benefitted from the knowledge accrued during outbreaks in other settings and selected an initial testing strategy based on risk rather than presentation with symptoms, as was used in many other countries. This strategy had positive public health implications for Uganda and allowed detection of cases that would otherwise have been missed and could potentially have escalated community transmission. Recent data has shown that much SARS-CoV-2 transmission occurs from pre-symptomatic patients [14–18], suggesting that the earlier an infected person can be detected – even an asymptomatic case – the more effectively transmission can be prevented [19]. Our transmission data appear to reflect this, with only five transmission chains identified from the 54 confirmed cases. The use of a primarily risk-based rather than primarily symptom-based testing strategy also likely accounts for why, as of early May, Uganda still had fewer cumulative cases than its neighboring countries [20].

Similarly, none of the case-persons developed severe disease or died. This contrasts with the outcomes of initial cases from most other countries [8, 21–24]. This is also likely due to differential case ascertainment approach used in Uganda, compared with other countries. The majority of case-persons in this evaluation were unlikely to have been identified without active, targeted, risk-based testing. This is consistent with accruing data from around the globe demonstrating that an even higher proportion of cases than initially suspected are asymptomatic or are minimally symptomatic [25–27]. The relatively young age of most case-persons in Uganda may also have contributed to the outcomes, however. Severity of the disease is known to increase with advanced age and co-morbidities [8, 28, 29]. The mean age of our case-persons was 35 years. In contrast, in Italy, where the case-fatality rate was 12% in mid-March 2020, the median age of the COVID-19 patients was 64 years [30, 31]. Although approximately two-thirds of the case-persons were male, this is likely a result of a gender bias in incoming travelers, many of whom were businessmen, rather than a meaningful epidemiologic pattern specific to Uganda.

The main source of the outbreak in Uganda was returnees entering the country through Entebbe airport. At the time the first case-person was identified in Uganda, having traveled from Dubai, the United Arab Emirates was not recognized as a ‘high-risk’ country. Subsequently, many other travelers from Dubai to Uganda, as well as to other countries, were recognized as being infected [32]. Understanding the risk posed by travelers from specific countries, especially in the face of high proportions of persons with asymptomatic SARS-CoV-2 infection and heterogeneous test availability and testing strategies, is a challenge. Countries that want to maintain a high degree of risk management – particularly those that manage to control transmission after an initial SARS-CoV-2 outbreak - may want to consider mandatory testing for all travelers at points of entry.

With the closure of the airport for commercial travel on March 21, 2020, the risk for SARS-CoV-2 infection in Uganda has shifted to other points of entries, at land borders. Porous borders in particular pose a major risk in Uganda, as they did with Ebola [33]. Focusing of strict border security and screening interventions, including testing of all persons crossing the border, is critical to the continued protection of Uganda. Identification of community-based approaches to ensure identification and testing of persons traveling through informal crossings will also be critical. For a coordinated strategy across East African regional member states to the COVID-19 pandemic, such as uniform application of testing strategies, could also facilitate improved control in the region.

Despite Uganda’s efforts to prevent and control this pandemic, the high prevalence of asymptomatic infections will present a major challenge to continuing to keep the disease at bay. Traditional public health disease control and prevention strategies rely heavily on early disease detection, usually through identification of symptomatic persons, to contain spread. The fact that only one patient in this series had all the ‘typical’ features [7] of COVID-19 disease suggests that the Ministry of Health will need to rely on a high index of suspicion for the disease among the high-risk populations, regardless of their symptomatology. Consideration should be placed on routine testing of high-risk groups, even when asymptomatic, as well as mass community testing in priority settings if community transmission is suspected. Including asymptomatic individuals, especially for enclosed populations (schools, prisons, refugee settlements), into the screening algorithm for SARS-CoV-2 infection will require scaling-up and subsequent decentralization of testing in the country. Furthermore, these data support expanded symptom screening to improve early detection of cases in Uganda, given that a significant proportion of the symptomatic case-persons had atypical presentations. Delayed diagnosis, often as a result of few or no symptoms, are contributing factors to sustained community transmission of influenza-like illnesses [34, 35]. While few transmissions occurred in our small case series, none were identified from asymptomatic persons. Continued intensive contact tracing, as shown in other places [36, 37], will be needed to continue to control the epidemic in Uganda.

Our findings are subject to some limitations. We analyzed data from only 54 case-persons in a setting of low community transmission. This may not give comprehensive understanding of the disease or the trajectory of the epidemic in Uganda. In addition, little community testing was occurring at the time outside of contact tracing testing, meaning that we may have under-ascertained cases.

## Conclusion

The first 54 case-persons in Uganda with SARS-CoV-2 infection were primarily imported and had asymptomatic or mild disease. Transmission was low, even from symptomatic persons; asymptomatic persons did not transmit infection. Targeted testing interventions and other interventions implemented in Uganda enabled detection of these infected persons and likely enabled early disease control in Uganda in the setting of the initial phase of COVID-19 pandemic.

## Recommendations

The Ministry of Health should consider intensifying routine and systematic screening of travelers and at-risk persons and emphasize thorough contact tracing. Increasing targeted testing among persons with a wider range of symptoms than is typically considered for COVID-19 should also be undertaken. Uganda must strengthen control measures at the points of entry to include routine and mandatory screening for SARS-CoV-2 for all travelers regardless of their symptomatology or origin, to minimize the risk of importation of the disease with subsequent community transmission.

## Data Availability

The data sets and the reports that support this write up belong to the MoH Uganda. For reasons of confidentiality, the datasets and reports are not publicly available. However, datasets and the reports could be availed upon reasonable request and with permission from the MoH Uganda.

## Abbreviations

COVID-19: Coronavirus Disease
HIV: Human Immunodeficiency Virus
IQR: Inter-quartile Range
MoH: Ministry of Health
NRH: National Referral Hospital
RRH: Regional Referral Hospital
SARS-CoV-2: Severe Acute Respiratory Syndrome Coronavirus 2
SD: Standard Deviation
UVRI: Uganda Virus Research Institute
